# The concentrations of bone calcium, phosphorus and trace metal elements in elderly patients with intertrochanteric hip fractures

**DOI:** 10.3389/fendo.2022.1005637

**Published:** 2022-12-13

**Authors:** Shangjin Lin, Cong Chen, Xiaoxi Cai, Fengjian Yang, YongQian Fan

**Affiliations:** Department of Orthopeadic, Huadong Hospital Affiliated to Fudan University, Shanghai, China

**Keywords:** trace element, bone metabolism, EDX, zinc, copper

## Abstract

**Introduction:**

Trace metal elements may play a crucial role in bone mineralization and metabolism. However, the quantification of trace element concentrations in human bone tissue has received little attention.

**Materials and methods:**

Bone tissue samples were collected from 55 elderly patients (15 males and 40 females) with intertrochanteric hip fractures. The calcium, phosphorus, manganese, iron, copper, and zinc concentrations in the cortical bone zone, cancellous bone zone, and junction zone between cortical and cancellous bone were determined by energy-dispersive X-ray fluorescence (EDX). The differences in trace element concentrations in the three regions were compared, and the correlation between gender and bone trace element contents of the bones was analyzed using the Kruskal-Wallis’s test. The correlation between age, body mass index (BMI), and bone calcium, phosphorus concentrations, and trace elements in three bone zones was determined using Spearman correlation analysis.

**Results:**

The Kruskal-Wallis test showed no difference in bone phosphorus concentration among the three regions. In contrast, the difference in the concentrations of bone calcium and four metal elements was statistically significant (*P*<0.01). In addition, no statistical differences were observed in the concentrations of trace elements among the three regions in elderly male and female patients. Spearman correlation analysis showed a strong negative correlation between bone calcium and phosphorus in three bone regions (r=-0.999, -0.95, -0.998, *P* < 0.01) and a significant positive correlation between trace metal elements in the cancellous bone zone. In the junction zone, the BMI showed a strong positive correlation with bone calcium content (r=0.347, *P*=0.009) and a significant negative correlation with phosphorus content (r=-0.349, *P*=0.009).

**Conclusion:**

Bone calcium and phosphorus were the main components of hydroxyapatite, and these two elements accounted for the majority of bone mineral salts. Trace metal elements are essential for bone metabolism and specific synergistic interactions. BMI may be associated with bone calcium and phosphorus contents in elderly patients with osteoporosis.

## Introduction

Bone tissue is composed of organic and inorganic substrates. Calcium and phosphorus are the main components of hydroxyapatite crystals, and calcium and phosphorus concentrations determine bone mass and mineral density (1). Trace metal elements are essential for bone mineralization and metabolism, with manganese, iron, copper, and zinc having the most impact on bone growth and development. As a cofactor of many hydrolases and transferases, manganese promotes the synthesis of bone minerals and cartilage mucopolysaccharides, which are essential in bone formation and chondrogenesis (2, 3). Iron is the most abundant metal trace element in the human body (4). Iron deficiency impairs ossein synthesis, which decreases bone strength and toughness. It also disrupts vitamin D3 activation and osteogenesis, reducing bone mass and strength (5). However, in recent years, researchers have speculated that excess iron might cause abnormal bone metabolism and mineralization activities, which may cause bone absorption and oxidative stress, reduce the biomechanical properties of bone, and increase the risk of fracture (6). Copper is an essential trace element for bone growth and calcium mineralization (7). It promotes the cross-linking of collagen and elastin to form bone matrix components. Copper can also stimulate the proliferation and differentiation of bone marrow mesenchymal stem cells into osteoblasts to promote new bone formation and mineralization (8). Zinc is most closely linked to bone metabolic activities (9). Zinc participates in the synthesis of bone-derived alkaline phosphatase and the regulation of vitamin D3 activity and function. In addition, zinc promotes the synthesis of the organic bone matrix by forming activation centers for collagenase and sulfating enzyme (10).

At present, trace elements in bone tissue have mainly been analyzed in animals. Some studies have determined the concentrations of trace elements by analyzing human tissues such as serum, hair, and urine. However, few studies have reported on the concentration of inorganic elements in human bone tissues. In this study, bone tissue samples were obtained from elderly patients with intertrochanteric fractures of the femur. Bone calcium, phosphorus, and trace metal elements (Zn, Cu, Fe, Mn) were measured in the cortical bone region, cancellous bone region, and junction region by EDX. This research determined the concentration of these six inorganic elements in the elderly population. The differences in the concentrations of inorganic elements in different regions were compared, and the correlation between bone calcium, phosphorus, and trace metal elements was determined.

## Methods

### Study participants and collection of clinical data

Between September 2015 and June 2018, 55 elderly patients, including 15 males and 40 females, aged between 64 and 100 years, with an average age of 83.67 ± 9.33 years, were treated for intertrochanteric hip fractures caused by low-energy injury in the Department of Orthopedics, Huadong Hospital Affiliated to Fudan University. On the second day of hospitalization, 51 subjects underwent laboratory examinations, including measurement of hemoglobin, serum albumin, serum bone alkaline phosphatase (ALP), and serum calcium and phosphorus levels. All patients consented to participate in this study. The protocol for this study was approved by the Ethics Committee of Huadong Hospital, Affiliated with Fudan University (No. 2014K40). The research was conducted following the Strengthening the Reporting of Observational Studies in Epidemiology (STROBE) statement (see **Supplementary 1** STROBE Checklist). Inclusion criteria were as follows: 1) Patients treated with PFNA-II internal fixation for intertrochanteric femoral fracture; 2) Older than 60 years old; 3) Absence of chronic liver and kidney diseases, metabolic diseases, immune diseases, infections, and tumors; 4) Absence of systemic diseases such as pendulous pneumonia or heart failure occurred during the perioperative period.

### Collection of bone samples

All subjects underwent PFNA-II internal fixation for intertrochanteric hip fractures. Intramedullary implantation of PFNA-II required the placement of a blade nail with a diameter of more than 1 cm along the femoral neck. Therefore, the bone tissue occupied by the blade nail was removed using a proximal femoral screw drill bit before insertion. We creatively completed the modification of the proximal femoral screw drilling bit to a hollow bit (**Figure S1**). The whole extraction of cylindrical samples, including cortical and cancellous bone, was achieved during surgery without affecting the surgical procedure and the patient (**Figure S2**). After collection with the hollow drilling bit during the surgical procedure, the bone samples were immediately fixed in a paraformaldehyde fixator and stored in a 4°C refrigerator.

### EDX measurement

The bone tissue specimens were embedded in plexiglass and fixed by laser. The anchor point was cut with a diamond saw blade to expose the specimen surface, which was then finely polished. No contamination with trace elements was detected in the process of embedding and surface preparation of bone samples. The thickness of the bone specimen was maintained at 4–5mm to normalize attenuation during the sample preparation. The EDX experiment was set up as follows: the acceleration voltage was 40kV; each measurement time lasted 100s; the detection status was vacuum; the X-ray wavelength was 2 mm. When the polished plexiglass-embedded specimen was placed in the vacuum sample chamber of the Orbis analyzer, specimens with low magnification (10X) were first scanned to identify three distinct regions of bone tissue (**Figure S3A**). Once we have selected the region of bone tissue to measure, the detector used in the Orbis analyzer was applied to randomly select two points with high magnification (100X) for measuring the relative concentrations of six elements (**Figure S3B–D**). The Orbis analyzer focuses the incident X-ray beam to a spot of about 30μm in diameter for a higher energy flux and greater physical and analytical resolution. After scanning the specimens using an Orbis analyzer, the concentrations of six elements, including Ca, P, Fe, Mn, Zn, and Cu, in the cortical bone, cancellous bone, and junction zone were determined by EDX analyses. Two parts of each zone were randomly selected for measurement, and the average concentrations of element components were determined.

### Statistical analysis

The statistical analysis was performed using SPSS v23.0 (SPSS, Inc, Chicago, IL, USA). Firstly, statistical descriptions for the primary clinical data of 55 subjects and the concentrations of trace elements in bone tissues of 55 subjects in different zones were performed. The normal distribution for variables of trace element concentrations was checked. Since these variables were not normally distributed, the difference in trace element concentrations in the cortical bone, cancellous bone, and junction zone was compared using the Kruskal-Wallis test. The differences in calcium, phosphorus, and trace element levels in bone tissue between male and female patients were also compared using the Kruskal-Wallis test. The correlation between age, BMI, and the relative contents of bone calcium, phosphorus, and trace elements in three zones was determined by Spearman correlation analysis. Corresponding 95% confidence limits (CIs) were calculated with confidence interval estimation, and *P*< 0.05 was considered statistically significant.

## Results

### Basic clinical data of 55 subjects

All the study subjects of this study had intertrochanteric hip fractures caused by low-energy trauma, typical of osteoporosis. The clinical data for all the subjects at baseline are shown in **Table 1**. The study participants averaged 83.67 years (range 64–100 years) and had relatively normal weight (BMI 21.6 ± 2.27 kg/m^2^) (**Table 1**). In addition, the subjects had low hemoglobin levels (106.51 ± 17.14 g/l) and normal serum albumin levels (37.05 ± 4.35 g/l).

**Table 1:** Baseline characteristics of the 55 elderly patients in the research.

Characteristics	Mean	SD	Max	Min	Median	IQR
**BMI (kg/m2)**	21.6	2.27	25.4	17.6	21.5	19.5-23.4
**Age (years)**	83.67	9.33	100	64	84	81-89
**Albumin (g/l)**	37.05	4.35	48	23	37.7	35-40
**Serum calcium (mmol/l)**	2.17	0.13	2.6	1.8	2.2	2.1-2.3
**Serum phosphorus (mmol/l)**	1.02	0.17	1.42	0.68	1.01	0.9-1.15
**ALP (u/l)**	78.69	28.78	197	43	68	59-97
**Hemoglobin (g/l)**	106.51	17.14	140	72	111	93-119

### Comparison of bone calcium, phosphorus, and metal elements concentrations in different zones of bone tissue

**Figure 1:**
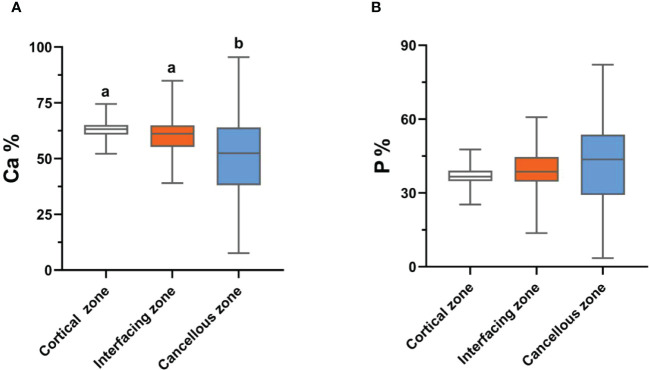
The distribution differences of bone calcium **(A)** and phosphorus **(B)** contents in the three zones. Values with different superscripts indicate a significant difference among groups based on Bonfferroni multiple range test (*P*< 0.05).

**Figure 2:**
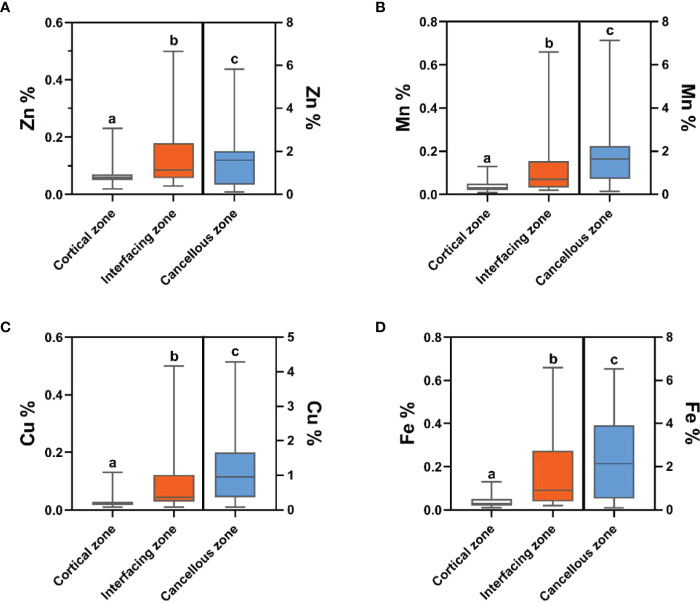
The distribution differences of bone zinc **(A)**, manganese **(B)**, copper **(C)** and iron **(D)** contents in the three zones. Trace element contents in cortical zone and interfacing zone are referenced to the left-hand Y axis, while trace element contents in cancellous zone are referenced to the right-hand Y axis. Values with different superscripts indicate a significant difference among groups based on Bonfferroni multiple range test (*P*< 0.05).

**Figure 3:**
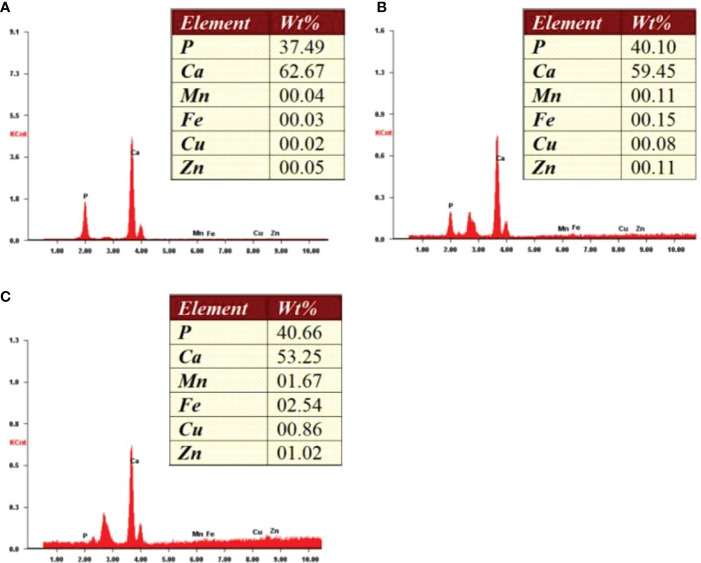
Energy-dispersive X-ray spectroscopy spectra of different zones from one bone tissue sample. **(A)** Cortical zone; **(B)** Interfacing zone; **(C)** Cancellous zone.

Bone inorganic element concentrations of calcium, phosphorus, manganese, iron, copper, and zinc among cortical bone zone, cancellous bone zone, and junction zone analyzed in different zones of bone tissue are shown in **Table 2**. The proportion of bone calcium concentration in the cortical, junction, and cancellous bone zone decreased sequentially, and the difference was statistically significant (*P*=0.004) (**Figure 1A**). The phosphorus concentration in bones was highest in the cancellous bone zone, lowest in the cortical bone zone, and moderate in the injunction zone. However, their difference was not statistically significant (**Figure 1B**). The Kruskal-Wallis test results revealed that differences in trace metal elements distribution in the three zones were statistically significant (*P* < 0.001) (**Figure 2**). Besides, the Energy-dispersive X-ray spectroscopy spectra of different zones of the same bone tissue sample are shown in **Figure 3**. The proportion of trace metal elements in the cancellous bone zone was the largest, followed by the junction zone, and was lowest in the cortical bone zone, as shown in **Table 3**.

**Table 2:** Bone inorganic element concentrations of calcium, phosphorus, and metal elements in different zones.

Bone region	Element	Mean	SD	SED	Median	IQR
**Cortical zone**	**Ca(%)**	62.56	4.32	0.58	63.19	60.75-65.07
	**P(%)**	37.27	4.35	0.59	36.63	34.75-39.11
	**Mn(%)**	0.033	0.022	0.0029	0.03	0.02-0.04
	**Fe(%)**	0.039	0.022	0.0029	0.03	0.02-0.05
	**Cu(%)**	0.024	0.018	0.0025	0.02	0.02-0.03
	**Zn(%)**	0.061	0.029	0.0039	0.06	0.05-0.07
**Cancellous zone**	**Ca(%)**	53.66	23.49	3.17	52.39	38.18-66.43
	**P(%)**	38.59	21.46	2.89	42.69	27.26-53.48
	**Mn(%)**	1.67	1.51	0.2	1.28	0.46-2.21
	**Fe(%)**	3.1	2.92	0.39	2.86	0.61-4.35
	**Cu(%)**	1.25	1.57	0.21	0.88	0.24-1.44
	**Zn(%)**	1.67	1.72	0.23	1.46	0.42-2.01
**Interfacing zone**	**Ca(%)**	60.29	9.03	1.22	61.13	55.22-64.96
	**P(%)**	39.17	9.19	1.24	38.68	34.61-44.62
	**Mn(%)**	0.099	0.11	0.014	0.07	0.03-0.14
	**Fe(%)**	0.2	0.22	0.029	0.12	0.04-0.33
	**Cu(%)**	0.096	0.12	0.017	0.03	0.02-0.11
	**Zn(%)**	0.14	0.13	0.018	0.09	0.06-0.21

**Table 3:** Comparison of bone calcium, phosphorus, and metal elements concentrations in different zones.

Element	Cortical zone	Interfacing zone	Cancellous zone	H value	P value
	Median (P25,P75)				
**Ca (%)**	63.19 (60.75-65.07)	61.13 (55.22-64.96)	52.39 (38.18-66.43)	15.54	**0.004**
**P(%)**	36.63 (34.75-39.11)	38.68 (34.61-44.62)	42.69 (27.26-53.48)	2.357	0.308
**Mn(%)**	0.03(0.02-0.04)	0.07(0.03-0.14)	1.28(0.46-2.21)	95.85	**<0.001**
**Fe (%)**	0.03 (0.02-0.05)	0.12 (0.04-0.33)	2.86 (0.61-4.35)	114.5	**<0.001**
**Cu (%)**	0.02 (0.02-0.03)	0.03 (0.02-0.11)	0.88 (0.24-1.44)	74.92	**<0.001**
**Zn (%)**	0.06 (0.05-0.07)	0.09 (0.06-0.21)	1.46 (0.42-2.01)	86.33	**<0.001**

Bold indicate statistically significant findings.

### Comparison of bone calcium, phosphorus, and trace element contents between elderly male and female patients in different zones of bone tissue

The differences in the proportions of calcium, phosphorus, and trace metal elements between male and female patients in the three regions of bone tissue are presented in **Table 4**. Unfortunately, there was no significant difference in the relative contents of bone calcium, phosphorus, and trace metal elements in the three regions between the two groups.

**Table 4:** Differences in the relative contents of bone calcium, phosphorus and trace metal elements between elderly male and female patients.

Element	Female (n=40)	Male (n=15)	Z Value	*P* Value
	Median, IQR	Median, IQR		
Cortical zone				
** Ca(%)**	63.98, 60.98-60.06	62.55, 58.87-65.14	0.95	0.35
** P(%)**	35.86, 34.73-38.85	37.37, 34.75-40.99	0.96	0.34
** Mn(%)**	0.03, 0.02-0.04	0.03, 0.02-0.05	0.29	0.77
** Fe(%)**	0.03, 0.02-0.048	0.04, 0.02-0.06	0.75	0.45
** Cu(%)**	0.02, 0.01-0.03	0.02, 0.01-0.03	0.53	0.59
** Zn(%)**	0.06, 0.05-0.07	0.05, 0.04-0.06	1.48	0.14
Cancellous zone				
** Ca(%)**	53.12, 38.75-66.19	51.12, 28.89-69.72	0.652	0.51
** P(%)**	37.99, 20.65-53.28	46.63, 28.74-53.92	0.66	0.51
** Mn(%)**	1.21, 0.49-2.17	1.78, 0.45-2.34	0.46	0.64
** Fe(%)**	2.99, 0.92-4.24	1.82, 0.45-4.92	0.47	0.64
** Cu(%)**	0.91, 0.25-1.72	0.45, 0.08-1.19	0.96	0.34
** Zn(%)**	1.37, 0.43-2.06	1.67, 0.34-1.89	0.01	0.99
Interfacing zone				
** Ca(%)**	61.16, 55.25-64.85	60.78, 55.09-67.41	0.13	0.89
** P(%)**	38.69, 34.99-44.59	38.67, 32.33-44.74	0.21	0.83
** Mn(%)**	0.06, 0.03-0.14	0.08, 0.04-0.16	1.29	0.19
** Fe(%)**	0.08, 0.04-0.32	0.14, 0.05-0.33	0.81	0.42
** Cu(%)**	0.03, 0.02-0.88	0.08, 0.01-0.18	0.25	0.81
** Zn(%)**	0.08, 0.05-0.17	0.13, 0.07-0.25	0.91	0.36

### Correlation analysis of bone calcium, phosphorus, and metal elements concentrations in different zones of bone tissue

To clarify the interdependence of inorganic elements in bone tissue, correlation analysis between calcium, phosphorus, and metal elements was performed using Spearman’s correlation test; the results are shown in **Table 5**. In the cortical bone zone, bone calcium concentration was strongly and negatively correlated with bone phosphorus concentration (r=-0.999, *P*<0.01), while bone zinc element concentration was strongly and positively correlated with bone calcium content (r=0.609, *P*<0.01), but negatively correlated with phosphorus concentration (r=-0.617, *P*<0.01). Besides, there was a strong positive correlation between bone manganese and bone iron (r=0.308, *P* < 0.05), and between bone manganese and bone copper (r= 0.313, *P* < 0.05).

**Table 5:** Correlation of bone calcium, phosphorus, and metal elements concentrations in different zones of bone tissue.

Element	Ca	P	Mn	Fe	Cu	Zn
Cortical zone						
** Ca**	1					
** P**	-0.999^**^	1				
** Mn**	-0.082	0.071	1			
** Fe**	-0.058	0.048	0.308^*^	1		
** Cu**	0.019	-0.025	0.313^*^	0.23	1	
** Zn**	0.609^**^	-0.617^**^	-0.024	0.17	0.108	1
Cancellous zone						
** Ca**	1					
** P**	-0.95^**^	1				
** Mn**	-0.581^**^	0.377^**^	1			
** Fe**	-0.518^**^	0.285^*^	0.686^**^	1		
** Cu**	-0.406^**^	0.183	0.689^**^	0.612^**^	1	
** Zn**	-0.329^*^	0.065	0.695^**^	0.657^**^	0.82^**^	1
**Interfacing zone**						
** Ca**	1					
** P**	-0.998^**^	1				
** Mn**	0.045	-0.079	1			
** Fe**	0.046	-0.077	0.739^**^	1		
** Cu**	0.219	-0.246	0.577^**^	0.566^**^	1	
** Zn**	0.437^**^	-0.468^**^	0.763^**^	0.623^**^	0.679^**^	1

Bold indicate statistically significant findings;* means P<0.05;** means P<0.01;*** means P<0.001.

In the cancellous bone zone, bone calcium had a significant negative correlation with all other elements (*P*<0.01). In contrast, bone manganese strongly and positively correlated with bone iron (r=0.686, *P*<0.01), copper (r=0.689, *P*<0.01), and zinc content (r=0.695, *P*<0.01). Bone iron was also significantly positively correlated with bone copper and bone zinc (r=0.612, 0.659, *P*<0.01). Similarly, there was a strong positive correlation between the concentration of bone copper and zinc element (r=0.682, *P*<0.01).

In the junction zone between cortical and cancellous bone, there was a strong negative correlation between calcium and phosphorus levels. Zinc concentration in bones was strongly correlated with the other five elements (Ca, P, Mn, Fe, and Cu), with correlation coefficients of 0.437, -0.468, 0.763, 0.623, and 0.679, respectively (*P* < 0.01). Similarly, there was a strong positive correlation (r=0.739, 0.577, *P*<0.01) between the concentration of manganese in bones and iron and copper contents in the bones. Furthermore, the bone iron concentration was also positively correlated with the bone copper concentration (r=0.566, *P*<0.01).

### Correlation analysis of age, BMI, and bone calcium, phosphorus, and trace metal elements contents in different zones of bone tissue

**Figure 4:**
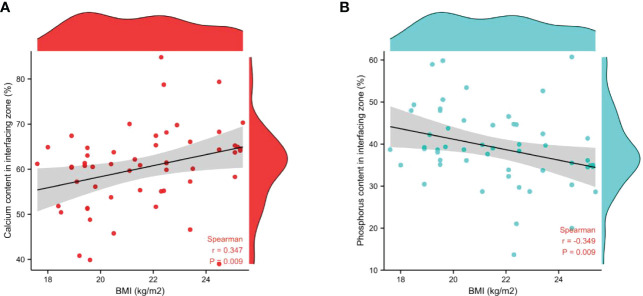
Correlation between BMI and the relative contents of bone calcium **(A)** and phosphorus **(B)** in interfacing zone.

The associations between age, BMI, and bone calcium, bone phosphorus, and trace element levels in different regions were determined using Spearman’s correlation test. There was no significant relationship between age, BMI, and level of bone trace metal elements across the three bone regions (**Table 6**). Nevertheless, a strong positive correlation between BMI and bone calcium content (r=0.347, *P*=0.009) and a significant negative correlation between BMI and bone phosphorus content (r=-0.349, *P*=0.009) were observed. The correlation between BMI and bone calcium and phosphorus contents in the interfacing zone is shown in **Figure 4**.

**Table 6:** Correlation of bone calcium, phosphorus, and metal elements concentrations with age and BMI.

	Age		BMI	
	Correlation coefficient	*P* Value	Correlation coefficient	*P* Value
Cortical zone				
** Ca**	0.22	0.11	0.19	0.17
** P**	-0.23	0.09	-0.2	0.15
** Mn**	0.01	0.94	0.12	0.37
** Fe**	-0.14	0.31	-0.03	0.83
** Cu**	0.11	0.45	0.067	0.63
** Zn**	0.049	0.72	0.072	0.6
Cancellous zone				
** Ca**	-0.13	0.33	-0.068	0.62
** P**	0.14	0.32	0.049	0.72
** Mn**	0.093	0.49	0.059	0.67
** Fe**	0.11	0.43	0.15	0.28
** Cu**	0.049	0.72	-0.098	0.48
** Zn**	-0.044	0.75	-0.065	0.64
Interfacing zone				
** Ca**	0.018	0.89	**0.347**	**0.009**
** P**	-0.029	0.84	**-0.349**	**0.009**
** Mn**	-0.059	0.67	-0.034	0.81
** Fe**	-0.017	0.9	0.094	0.49
** Cu**	0.18	0.19	0.017	0.91
** Zn**	0.044	0.75	0.094	0.49

Bold indicate statistically significant findings.

## Discussion

Calcium and phosphorus are the essential elements of hydroxyapatite in bone tissue, which directly reflect bone density and mineralization in the body (11). In recent years, the role of trace metal elements in the pathogenesis of osteoporosis has attracted much attention. Trace metal elements regulate and participate in bone formation and bone metabolism. Excessively high or low levels of trace metal elements in the body can affect the bone mineral formation and bone matrix synthesis, thereby promoting the development of osteoporosis (12). Consequently, this study directly measured the concentrations of calcium, phosphorus, manganese, iron, copper, and zinc in human bone tissue using EDX and compared the concentrations of metal trace elements in different zones of bone samples.

The accurate determination of inorganic elements in bone tissue is mainly determined by flame atomic absorption spectrometry (FAAS), inductively coupled plasma mass spectrometry (ICP-MS), and energy-dispersive X-ray fluorescence (EDX). This study used EDX analysis to determine the relative concentrations of inorganic elements in different zones of bone tissue since it was time-efficient and easy to operate. Besides, EDX analysis not only prevents the loss of trace elements in the original bone tissue but also measures the contents of inorganic elements in cortical bone, cancellous bone, and their junction zone with higher precision and accuracy than the other two methods (13, 14). Although this method can only measure the relative content of inorganic elements in bone tissue, the absolute content of bone elements is affected by many factors, such as gender, weight, age, height, race, and regional differences. Moreover, the measurements of absolute contents of bone elements can overestimate or underestimate the loss or gain of trace elements, which undermines the precision due to the inability to simultaneously measure inorganic element content in cortical bone, cancellous bone, and junction area of the same bone tissue.

The cortical bone zone of bone tissue predominantly consists of dense bone plates, which are plate-like structures with high mineral content. Therefore, cortical bone is mainly composed of calcium and phosphorus. In contrast, cancellous bone is a porous interwoven bone structure with needle-like or flaky trabeculae. In this study, Kruskal-Wallis test results revealed that the differences in the proportional distribution of the four trace metal elements in the three regions were statistically significant (*P* < 0.001). Mn, Fe, Zn, and Cu proportion was highest in the cancellous bone region and lowest in the cortical bone region. Brodziak-dopierala et al. (15) used FAAS to detect the contents of manganese and iron in different areas of hip joint bone tissues and found that manganese and iron were lowest in the cortical bone, while manganese content was the highest in articular cartilage. Iron content was the highest in the cancellous bone near the intertrochanter of the femur. Although the methods of quantitative detection of the element content were different, our results were consistent.

This study shows that trend in the levels of four trace metal elements in the cancellous bone zone and junction bone zone were as follows: Fe>Zn>Mn>Cu. Zaichick et al. (16) biopsied rib tissues of 80 cadaver cases and found that the trend in concentrations of trace metal elements in bone tissues was Fe>Zn>Cu>Mn by ICP-MS. Budis et al. (3) tested the contents of manganese, iron, and strontium in the cartilage and cancellous bone of the hip joint from 37 patients undergoing total hip replacement. The investigators found that iron content was the highest while manganese content was the lowest. Roczniak et al. (17) determined that the zinc level in knee bone tissues of 50 patients undergoing total knee arthroplasty by ICP-MS was significantly higher than the copper content. Regarding trace metal elements, including Zn, Cu, Fe, and Mn, the iron content was highest, followed by zinc. Increasing evidence shows that the iron content may gradually increase in the body with age, especially in patients with osteoporosis. Both serum iron level and bone iron content were significantly higher in osteoporosis group than those in the control group, while other trace metal elements decreased with age (16, 18). Herein, we found that manganese content was higher than copper content, conflicting Zaichick’s research findings. The difference in the findings might be attributed to the various methods used to measure the contents of trace elements and the difference in the age of the subjects. The content of copper and manganese must be confirmed in the later study using large samples, multiple age groups, and the same detection method.

The correlation analysis of inorganic elements showed a significant negative correlation between calcium content and phosphorus content in all zones (*P*<0.01), and the correlation coefficients in the cortical bone zone, junction bone zone, and cancellous bone zone were -0.999, -0.998, and -0.95, respectively. The findings were thought to be due to the high proportion of calcium and phosphorus among all elements. The closer the calcium and phosphorus proportions were to 100%, the stronger the negative correlation. We believe there is a certain degree of negative correlation between calcium and phosphorus elements. However, the specific correlation coefficient should be determined in future studies by measuring the absolute content of calcium and phosphorus elements. The results of correlation analysis between calcium, phosphorus and trace metal elements indicated a significant positive correlation between calcium and zinc content (r =0.609, 0.437, *P*<0.01) and a significant negative correlation between phosphorus and zinc content (r =-0.617, -0.468, *P*<0.01) in the cortical bone zone and junction zone. This correlation suggests that zinc plays a more critical role than other metal elements in bone mineralization and osteogenesis. Kuo et al. (19) measured inorganic element contents by ICP-MS in bone tissue samples from 77 Taiwanese and found a significant positive correlation between bone calcium and bone zinc. Zinc participates in synthesizing more than 200 enzymes in the body, including osteoblast-secreted alkaline phosphatase. Zinc is essential for regulating the function and activity of vitamin D3. Moreover, Zinc stimulates the initiation of gene apoptosis of mature osteoclasts and inhibits the differentiation of bone marrow precursor cells into osteoclasts (20). In addition, zinc also participates in bone mineralization and calcium salt deposition with calcium ions, which is considered an essential component of the calcified matrix (21).

Manganese content was strongly and positively correlated with the contents of iron, copper, and zinc in the cancellous and junction bone zone. This positive correlation indicated that manganese and the other three metal elements might synergistically stimulate bone metabolism and mineralization. Budis (3) also found a significant positive correlation between iron and manganese content in cortical and cancellous bone tissues (r =0.439, 0.653, *P* < 0.05, *P* < 0.001). Wang et al. (18) analyzed the serum manganese and zinc contents of 91 elderly male volunteers in Beijing and found a significant positive correlation between manganese and zinc content, with a correlation coefficient of 0.422. Likewise, Kuo (19) found a significant positive correlation between bone manganese and bone copper content, which was consistent with the findings of the study. In addition, we found that iron content in the cancellous bone region and junction region was significantly positively correlated with zinc and copper content (*P* < 0.01), while copper content was also significantly correlated with zinc content, with correlation coefficients of 0.82 and 0.679, respectively. Brodziak-dopierala (22) found a significant positive correlation between bone copper and zinc content in cancellous bone and articular cartilage, with correlation coefficients of 0.51 and 0.56, respectively. Also, there was a significant positive correlation between bone iron and manganese in cancellous bone and articular cartilage, with correlation coefficients of 0.61 and 0.55. Lanocha et al. (23) measured the content of copper and zinc by ICP-MS in the articular cartilage and cancellous bone tissue bone samples. The finding indicated a strong positive correlation between copper and zinc, with correlation coefficients of 0.57 and 0.47, respectively. Generally, our results showed statistically significant positive correlations between four trace metal elements. Meanwhile, the values of those correlation coefficients between trace metal elements were comparable to past findings. Trace metal elements, essential for bone growth and development, interact with the bone matrix and influence bone metabolism (24). Since little is known about the synergistic interactions between trace metal elements in the process of skeletal mineralization and resorption, the complexity of these interactions makes it extremely difficult to interpret the results. In the human body, these trace metal elements exist in the form of metals or compounds. Consequently, the interaction results suggest that ions with the same charge can alter each other’s effect.

In the research, we observed that BMI was positively correlated with bone calcium content, but negatively correlated with bone phosphorus content in the junctional region. This positive correlation of calcium suggests that in elderly individuals with a normal BMI, bone calcium content increases with BMI, thereby contributing to the maintenance of high bone mass. Previous studies have also found that BMI has a certain correlation with bone strength and bone mass. A pilot screening study for low bone mass in Singaporean women have found that the lower BMI may be associated with increased risk of low bone mass (25). In another cross-sectional study, it was also found that the lower BMI mediated osteoporosis and fragility fractures in patients at nutritional risk (26). Although these findings are consistent with the conclusions of this study, the relationship between BMI and bone calcium and phosphorus contents needs to be confirmed with larger population studies.

Although this study revealed many significant findings, there are still some shortcomings. First, our research subjects are mainly elderly patients with osteoporosis, and there was no non-osteoporotic control group. Therefore, the correlation between bone trace metal elements and osteoporosis could not be analyzed. Another limitation of our study is the relatively small sample size. Our findings must, thus, be validated by future studies with large samples size of individuals of various ages, sexes, and diseases.

## Conclusion

In conclusion, bone calcium and phosphorus are the primary components of hydroxyapatite, and these two elements comprise the majority of bone mineral salts. There was a strong positive correlation between bone zinc and calcium content and a strong negative correlation between bone zinc and phosphorus content. This difference in correlation suggests that zinc plays a role in regulating calcium and phosphorus metabolism. Significant positive correlations between these four trace metal elements suggest that trace elements, especially metallic ones, play a crucial role in bone metabolism and specific synergistic interactions. Furthermore, BMI may be associated with bone calcium and phosphorus contents in elderly patients with osteoporosis.

## Data availability statement

The original contributions presented in the study are included in the article/**Supplementary Material**. Further inquiries can be directed to the corresponding author.

## Ethics statement

The studies involving human participants were reviewed and approved by the informed consent was obtained from all patients in this clinical study, approved by the Ethics Committee of Huadong Hospital Affiliated to Fudan University (No. 2014K40). The patients/participants provided their written informed consent to participate in this study.

## Author contributions

YF and FY designed the research and revised the manuscript. SL and CC contributed to drafting the manuscript. XC and FY participated in data collection and analysis of data for the work. All authors contributed to the article and approved the submitted version.

## Supplementary material

Supplementary Figure 1 The modification of the proximal femoral screw drilling bit to hollow bit.

Supplementary Figure 2 Cylindrical bone samples including cortical and cancellous bone; **(A)**: cancellous bone zone; **(B)**: cortical bone zone.

Supplementary Figure 3 Microscopic image of bone tissue sample. **(A)** Low magnification image (10X); **(B)** High magnification image of cortical bone region; **(C)** High magnification image of cancellous bone region; **(D)** High magnification image of the junction region between cortical and cancellous bone.
