# Supplementary material for: The concentrations of bone calcium, phosphorus and trace metal elements in elderly patients with intertrochanteric hip fractures
Source: Front Endocrinol (Lausanne). 2022 Dec 13;13:1005637. doi: 10.3389/fendo.2022.1005637 (PMC9793898; doi:10.3389/fendo.2022.1005637)
Supplement: Supplementary Figure 1 — The modification of the proximal femoral screw drilling bit to hollow bit. [file Image_1.pdf]

Supplementary Figures

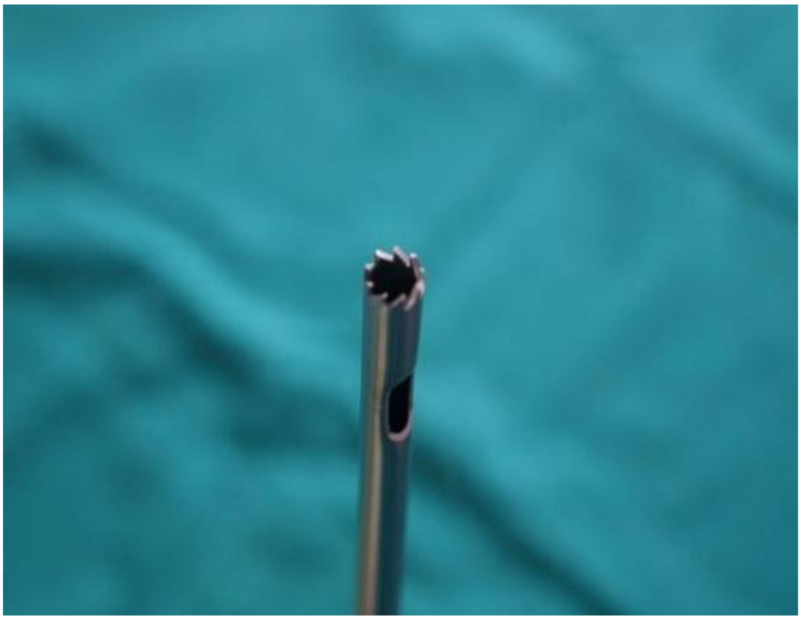

Figure S1. the modification of the proximal femoral screw drilling bit to hollow bit

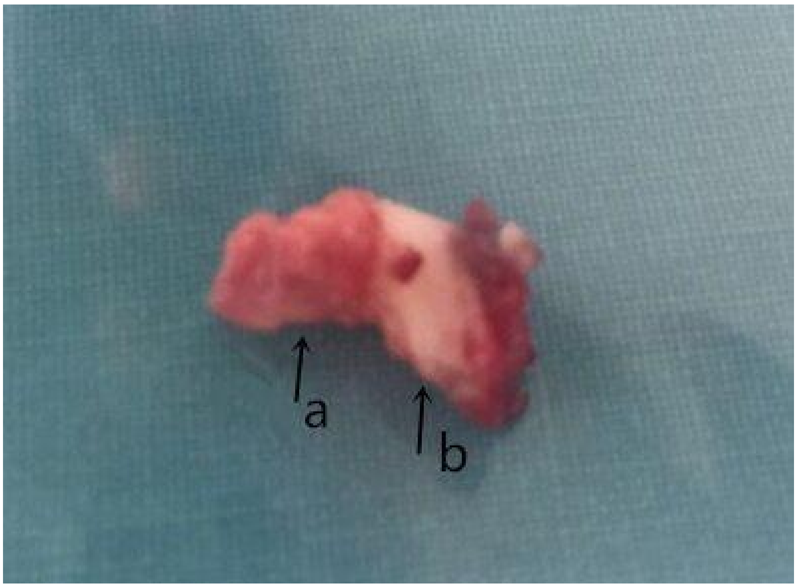


Figure S2. cylindrical bone samples including cortical and cancellous bone; **a**: cancellous bone zone; **b**: cortical bone zone

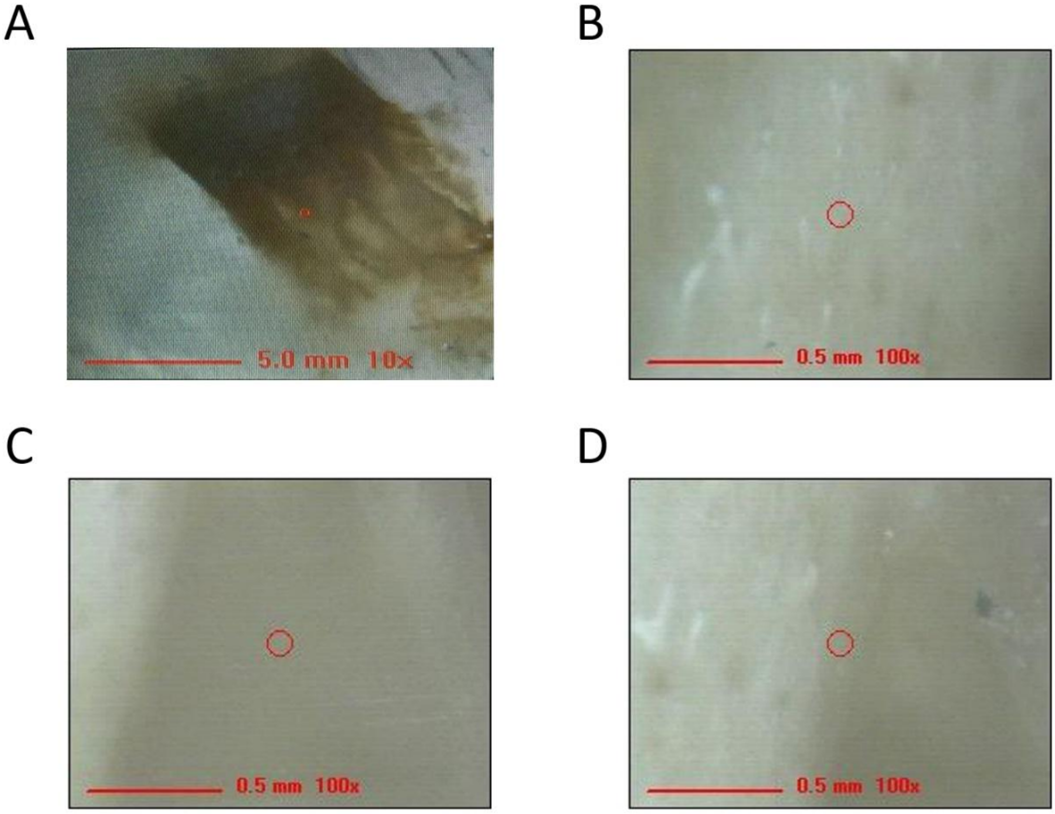


Figure S3. Microscopic image of bone tissue sample. (A) Low magnification image (10X); (B) High magnification image of cortical bone region; (C) High magnification image of cancellous bone region; (D) High magnification image of the junction region between cortical and cancellous bone.
